# Torsional resistance of additively, subtractively, and conventionally manufactured occlusal devices

**DOI:** 10.1007/s00784-025-06362-w

**Published:** 2025-05-06

**Authors:** Tina Maleki, Andrea Coldea, John Meinen, Marcel Reymus, Daniel Edelhoff, Bogna Stawarczyk

**Affiliations:** 1 Department of Prosthetic Dentistry, University Hospital, LMU Munich, Goethestrasse 70, 80336 Munich, Germany; 2 Department of Conservative Dentistry and Periodontology, University Hospital, LMU Munich, Goethestrasse 70, 80336 Munich, Germany

**Keywords:** Occlusal devices, 3D-printing, Torsion, Torque load, Angular rotation

## Abstract

**Objectives:**

To investigate the torque load (TL) and angular rotation (AR) of additively, subtractively and conventionally manufactured occlusal devices.

**Materials and methods:**

Specimens (*N* = 120) were fabricated from four additive manufacturing resins (GR-10 guide, ProArt Print Splint clear, V-Print Splint, V-Print Splint comfort), five subtractively (BioniCut, EldyPlus, ProArt CAD Splint clear, Temp Premium Flexible, Thermeo) and one conventionally manufactured (Pro Base Cold) materials. The TL and AR were tested initially (24 h, 37 °C, H_2_O) as well as after thermal cycling (5,000 thermal cycles, 5/55°C). Data were analyzed using Kolmogorov–Smirnov, one-way ANOVA, Scheffé post-hoc, t-test, chi-square and Ciba-Geigy table (*p* < 0.05).

**Results:**

Initially, the mean TL values ranged from 63.7 to 104 Ncm for additively, 39.2 to 265 Ncm for subtractively, and 204 Ncm for conventionally manufactured materials. The initial mean AR values were 41.7 to 143 deg for additively, 38.4 to 138 deg for subtractively, and 29.3 deg for conventionally manufactured materials. After thermal cycling, the mean TL values ranged from 45.7 to 88.1 Ncm for additively, 31.2 to 246 Ncm for subtractively, and 138 Ncm for conventionally manufactured materials. The mean AR values after aging ranged from 19.9 to 124 deg for additively, 48.1 to 131 deg for subtractively, and 19.5 deg for conventionally manufactured materials.

**Conclusions:**

The torsional resistance of additively, subtractively, and conventionally manufactured materials for occlusal devices varies and is affected by aging processes.

**Clinical Relevance:**

Material selection for occlusal devices should be guided by the clinical needs of the patients.

## Introduction

Occlusal devices are widely utilized in dental disciplines, serving multiple therapeutic purposes. In orthodontics, splints achieve tooth position changes, while in oral and maxillofacial surgery, splints are used for the treatment of fractures. Surgical guides assist in accurate and effective implantation. Patients who suffer from severe snoring (rhonchopathy) or sleep apnea wear splints at night to reduce their symptoms. The number of home bleaching splints on the market is also rapidly increasing. However, the most common clinical indication is the use of splints for patients with bruxism and craniomandibular dysfunction (CMD). Bruxism can be differentiated between sleep and awake bruxism [1]. Severe bruxism can lead to hypertrophy of the masseter and temporales muscles, abrasions of the enamel down to the dentin, resulting in a loss of vertical dimension of occlusion and extensive prosthetic treatment. Patients with CMD suffer from pain in the jaw, face, or neck area, may experience restricted jaw opening, deviations during jaw opening/closing, as well as noises in the temporomandibular joint. The primary goal of splint therapy for CMD patients is to minimize the pain symptoms by providing a balanced jaw alignment and prevent dysfunctional stresses to the temporomandibular joint, often in combination with manual physiotherapy [2]. The indication for the various splint types, e.g. Michigan splints, gelb splints, anterior bite plane splints, repositioning splints, distraction splints and aqualizer, depends strictly on the clinical situation [3]. Therefore, suspected diagnoses such as disc displacements with or without repositioning must be confirmed by MRI after thorough clinical examination. After diagnosis and planning, the dental impressions of the upper and lower jaw are taken. This can be done conventionally using impression materials such as alginate. Alternatively, a precise 3D model can be created digitally using intraoral scanners. A distinction is made between the chairside technique, where intraoral scanners are used directly, and labside digital methods, which involve scanning a physical impression or model in the laboratory. Traditionally, splints are fabricated using the injection technique with auto- or heat-polymerizing liquid-powder polymethylmethacrylate (PMMA) or the vacuum-forming technique with polyethylene discs [4, 5]. Despite its long-standing reliability, conventional manufacturing has several disadvantages, including residual monomer content, polymerization shrinkage, mixing errors, high time expense and laboratory costs [6, 7]. The monomer-to-polymer ratio, polymerization duration, and temperature depend on the dental technician. These factors influence the degree of conversion and, consequently, the mechanical properties [8]. Through computer-aided design (CAD) and computer-aided manufacturing (CAM) splints can be produced using either subtractive milling or additive 3D-printing techniques. These technologies offer advantages over the conventional method by providing easy access to patient-specific data and enabling a more time efficient fabrication of reproducible splints. Subtractively manufactured materials also show reduced polymerization shrinkage due to the high double-bond conversion rate of industrially produced PMMA discs [7, 8]. In subtractive milling, only two splints can be nested at once. This leads to significant tool wear and a high amount of unused blank material [9]. Through additively manufacturing, more splints can be produced simultaneously, depending on the size of the build platform and the printer. Top-down 3D-printers can print a much higher number of restorations compared to bottom-up 3D-printers. Additionally, material wear is significantly lower compared to the subtractive manufacturing since excess resin can be reused for the next print job. The Digital Light Projection (DLP) and Stereolithography (SLA) technology are the used methods for printing dental splints [8, 10]. Due to high intraoral forces (with bruxers producing forces between 450 N and 650 N, averaging around 380 N), splints are susceptible to fracture in clinical situations [8]. Therefore, the mechanical properties of splints are important. Recent studies primarily focus on hardness, wear, flexural properties, elastic modulus, fracture toughness, polishability, water sorption and solubility of additively, subtractively and conventionally manufactured occlusal devices [4–6, 10–21]. It is shown that their properties are significantly influenced by the composition of the material rather than the manufacturing technology [5, 10]. Also, the post-curing method, printer type, printing angle, printing direction and printing layer thickness affect the properties of additively manufactured splints [17, 22–24]. While a trend toward more flexible materials can be observed in the dental market, studies show that especially soft-type printed occlusal devices demonstrate lower properties regarding flexural strength, hardness values, wear, fracture resistance, water sorption and solubility [6, 15, 16]. It is observed that hard occlusal splints effectively decrease muscle activity and bite force transmitted to the teeth and temporomandibular joint discs compared to soft occlusal splints [25–27]. However, other studies show that soft splints better protect teeth from bending forces and lead to quicker symptom relief in CMD patients while also reducing salivary cortisol in bruxism sufferers and requiring less effort during clenching, as evidenced by reduced brain activity compared to hard splints [28–31]. The existing literature predominantly investigates bar, disc or rectangular specimen [11]. This study focuses on clinical splint geometries. By measuring the torque load and angular rotation of splints at failure in a torsional test, information about the resistance of a material to fracture and its threshold of plastic deformation can be obtained [32, 33]. Torque load refers to the rotational force applied until failure, while the angle of rotation measures the extent of deformation. The purpose of this in vitro study was to evaluate the effect of the material and artificial aging on torsional resistance by using a clinical splint geometry to identify the advantages and the limitations of different occlusal device materials. The tested null hypotheses were as follows: (a) material and (b) aging have no effect on torque load and angular rotation tested in this investigation.

## Materials and methods

**Fig. 1:**
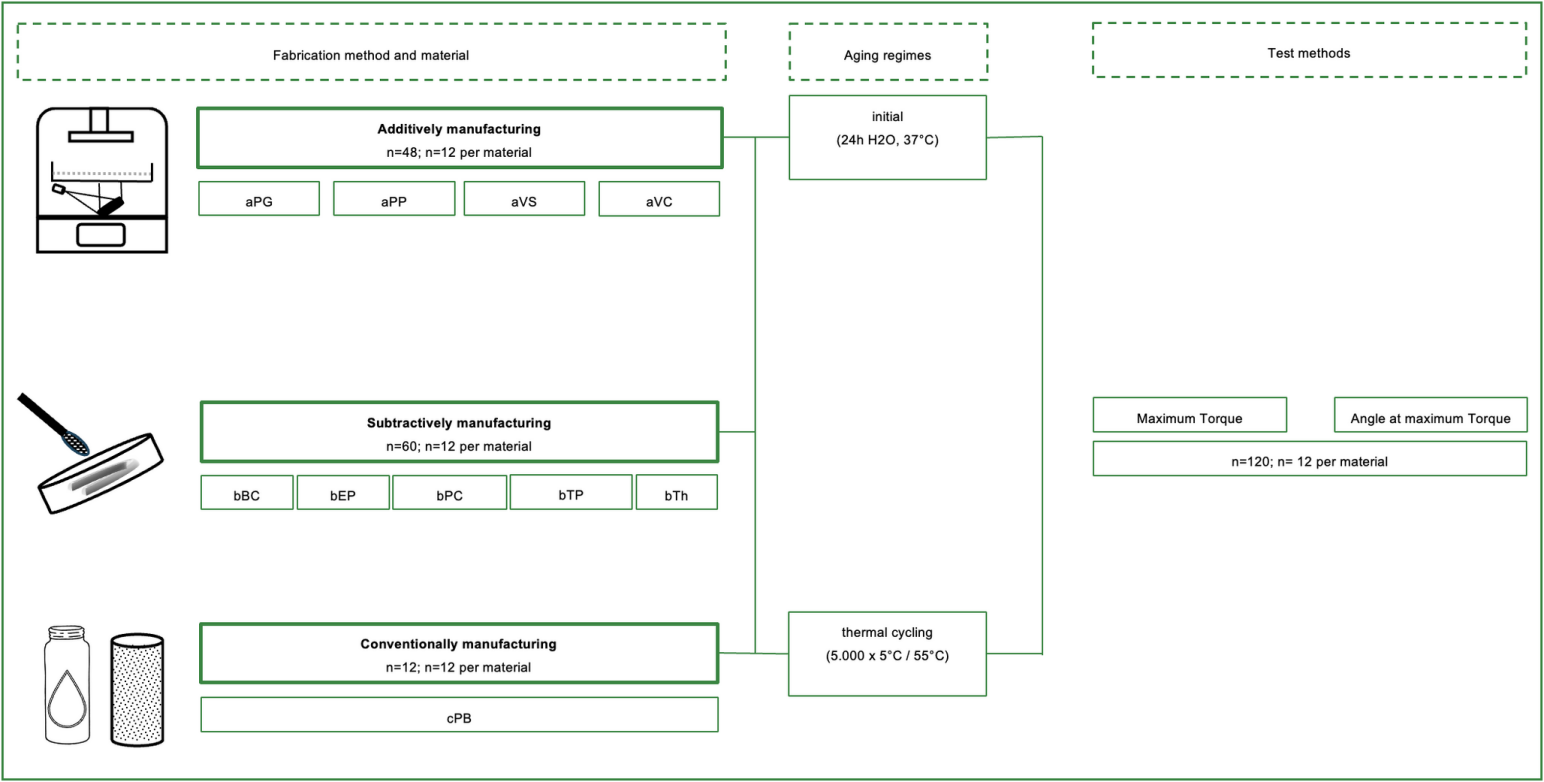
Study design

The occlusal devices were fabricated from four additive materials (GR-10 guide [aPG], ProArt Print Splint clear [aPP], V-Print Splint [aVS], V-Print Splint comfort [aVC]) (*n* = 48; *n* = 12 per material), five subtractive materials (BioniCut [bBC], EldyPlus [bEP], ProArt CAD Splint clear [bPC], Temp Premium Flexible [bTP], Thermeo [TH]) (*n* = 60; *n* = 12 per material) and one conventional material (Pro Base Cold [cPB]) (*n* = 12; *n* = 12 per material), and were then examined for their torsional resistance (Fig. 1; Table 1).

**Table 1:** The manufacturing types, abbreviations, product name, manufacturer, City, country, compositions, and lot numbers (Lot No.) of tested materials

Manufacturing type	Abbreviation	Product name	Manufacturer, city, country	Composition	Concentration %	Lot No.
additively manufacturing	aPG	GR-10 guide	pro3dure medical, Iserlohn, Germany	Esterification products of 4,4’- isopropylidene diphenol, ethoxylated, and 2- methylprop-2-enoic acid	80–100	030220221
				Diphenyl (2,4,6- trimethylbenzoyl) phosphine oxide	< 03	
	aPP	ProArt Print Splint clear	Ivoclar Vivadent, Schaan, Liechtenstein	Urethane acrylate oligomer	n.g.	Z02PY5
				Triethylene glycol dimethacrylate		
				Urethane dimethacrylate (UDMA)		
				Bis-glycidyl dimethacrylate(BisGMA)		
				Diphenyl(2,4,6-trimethylbenzoyl) phosphine oxide		
	aVS	V-Print Splint	VOCO, Cuxhaven, Germany	Polyester dimethacrylate	50–100	2,202,641
				Ethoxylated bisphenol-A dimethacrylate (Bis-EMA)	25–50	
				Triethylene glycol dimethacrylate	5–10	
				Hydroxypropylmethacrylate	5–10	
				Diphenyl (2,4,6-trimethylbenzoyl) phosphine oxide	≤ 2.5	
				Butylated hydroxytoluene (BHT)	≤ 2.5	
	aVC	V-Print Splint comfort		Aliphatic acrylate	25–50	2,209,436
				Triethylene glycol dimethacrylate	5–10	
				Diphenyl(2,4,6-trimethylbenzoyl) phosphine oxide	≤ 2.5	
subtractively manufacturing	bBC	BioniCut	Bredent, Senden, Germany	Polyoxymethylene (POM -C); pigments	n.g.	513,005 (height 20) 523,543 (height 25)
	bEP	EldyPlus	DentalPlus, Samerberg, Germany	Polyethylene terephthalat (PET-G); glykol; ethanol	n.g.	21ELGK26
	bPC	ProArt CAD Splint clear	Ivoclar Vivadent	Polymethylmethacrylate (PMMA)	> 99	YB3KR3
	bTP	Temp Premium Flexible	Zirkonzahn, South Tyrol, Italy	Thermoplastic polycarbonate (PC)	n.g.	16,261
	bTH	Thermeo	pro3dure medical	Polyethyl methacrylate, homopolymer	> 90	240,120,221
				1,2-Cyclohexanedicarboxylic acid diisononyl ester	< 10	
conventionally manufacturing	cPB	Pro Base Cold	Ivoclar Vivadent	Powder Polymethyl methacrylate (PMMA) dibenzoyl peroxide	< 95 1-2.5	YB380P (powder) Z032DH (liquid)
				Liquid Methyl methacrylate 1,4-butanediol dimethacrylate	60–100 3–5	

### Occlusal device Preparation

**Fig. 2:**
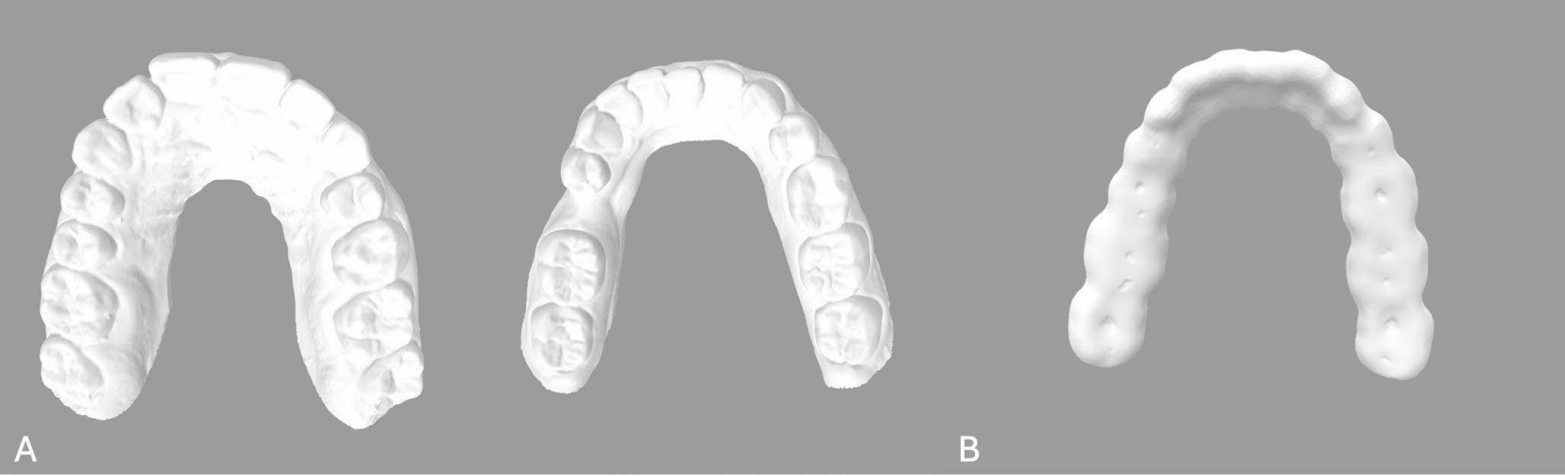
stl master data sets of the upper and lower jaw (**A**). Occlusal device (**B**)

To design an occlusal device upper and lower jaw plaster models with an intermaxillary gap in region 35 were used. The plaster models were scanned (Ceramill Map400, Amann-Girrbach, Koblach, Austria) and the standard triangle language (stl) master data sets of the upper and lower jaw were generated (Fig. 2A).

An occlusal device for the lower jaw was designed utilizing exoCAD software (DentalCAD, exocad, Darmstadt, Germany). A virtual tooth region 35 was constructed in the interocclusal space using the software. A minimum occlusal layer thickness of 2 mm and a spacer of 50 μm were used (Fig. 2B).

#### Additively manufactured occlusal devices

**Fig. 3:**
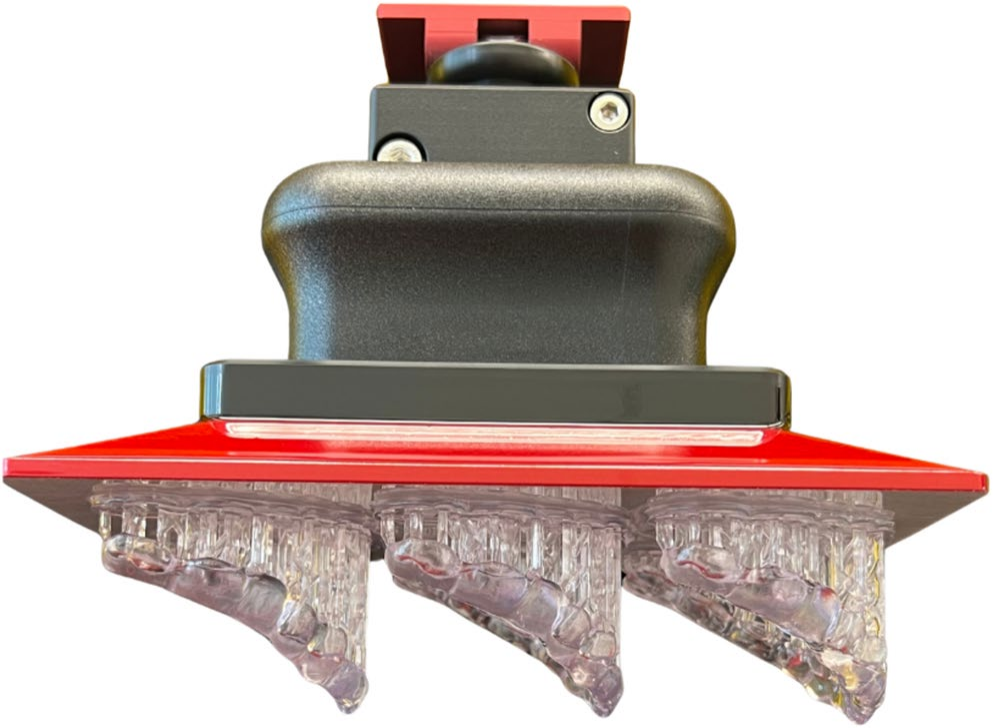
Additively manufactured specimens

The occlusal devices were additively manufactured using Digital Light Processing (trix^TM^print^2^, Dekema, Freilassing, Germany; PrograPrint PR5, Ivoclar, Schaan, Liechtenstein) (Table 2). The specimens were placed at a 45-degree angle with the occlusal surface away from the build platform and the support structures were automatically generated (trix^TM^CAD, Dekema) (Fig. 3). The post-processing steps were performed according to the manufacturer’s instructions (Table 2).

**Table 2:** Printer used and the post-processing of the additively manufactured materials tested

Material	DLP 3D-printer	Cleaning	Postpolymerization
aPG	trix^TM^print^2^, Dekema, Freilassing, Germany	4 min in IPA ≥ 97% (SAV Liquid Production, Flintsbach am Inn, Germany) in an ultrasonic bath (Sonorex Super RK1022; Bandelin electronic)	2 × 1000 flashes (Otoflash G171, NK-Optik, Baierbrunn, Germany)
aPP	PrograPrint PR5, Ivoclar Vivadent, Schaan, Liechtenstein	10 min pre-cleaning 5 min cleaning in IPA ≥ 97% (SAV Liquid Production) in PrograPrint Clean (Ivoclar Vivadent)	90 s in PrograPrint Cure (Ivoclar Vivadent)
aVS	trix^TM^print^2^, Dekema	3 min pre-cleaning 2 min cleaning in IPA ≥ 97% (SAV Liquid Production) in an ultrasonic bath (Sonorex Super RK1022; Bandelin electronic)	1 × 2000 flashes 2 min cooling 1 × 2000 flashes (Otoflash G171, NK-Optik)
aVC		5 min pre-cleaning 3 min cleaning in IPA ≥ 97% (SAV Liquid Production) in an ultrasonic bath (Sonorex Super RK1022; Bandelin electronic)	1 × 2000 flashes 2 min cooling 1 × 2000 flashes (Otoflash G171, NK-Optik)

#### Subtractively manufactured occlusal devices

**Fig. 4:**
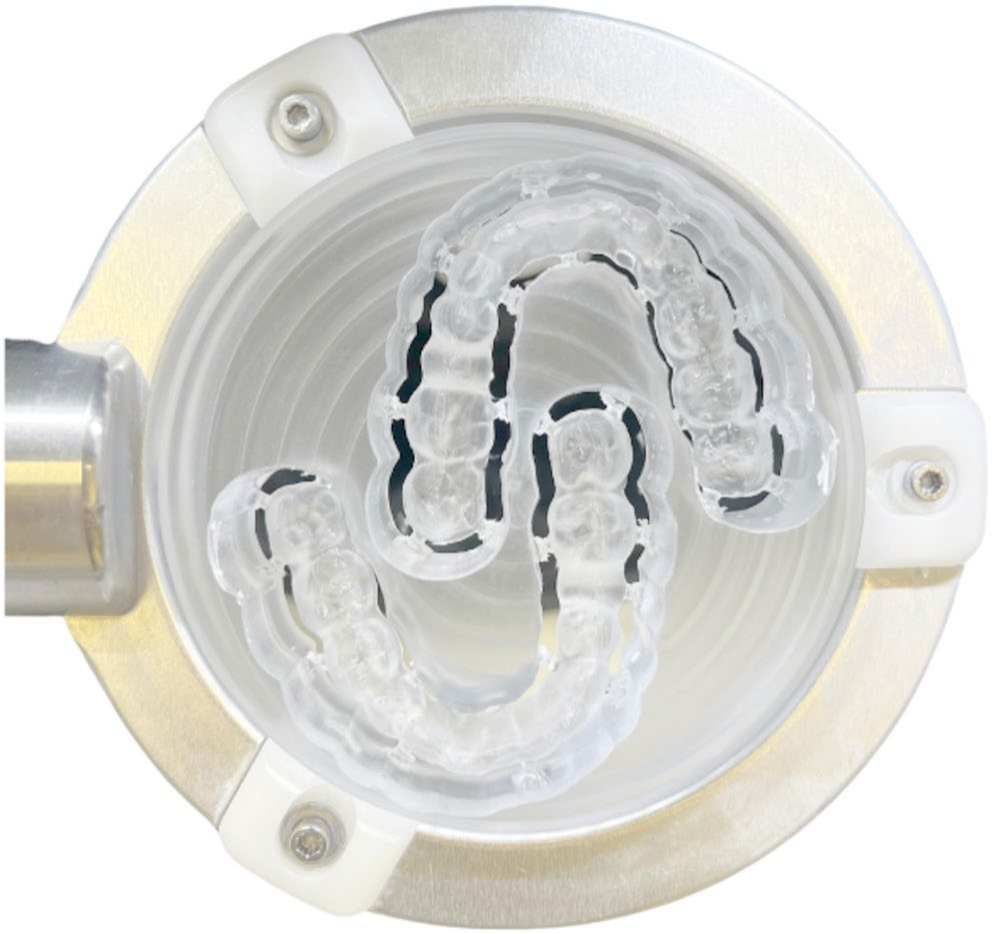
Subtractively manufactured specimens

The splints were nested into blanks (Ceramill Mind, Amann-Girrbach) and milled (Ceramill Motion II, Amann-Girrbach; breCAM.cutter TP, bredent, Senden, Germany) (Fig. 4). The finished splints were removed using a diamond disc (D2014, Komet, Lemgo, Germany) and the connectors were machined to achieve a smooth surface (H251EL, Komet).

#### Conventionally manufactured occlusal devices

**Fig. 5:**
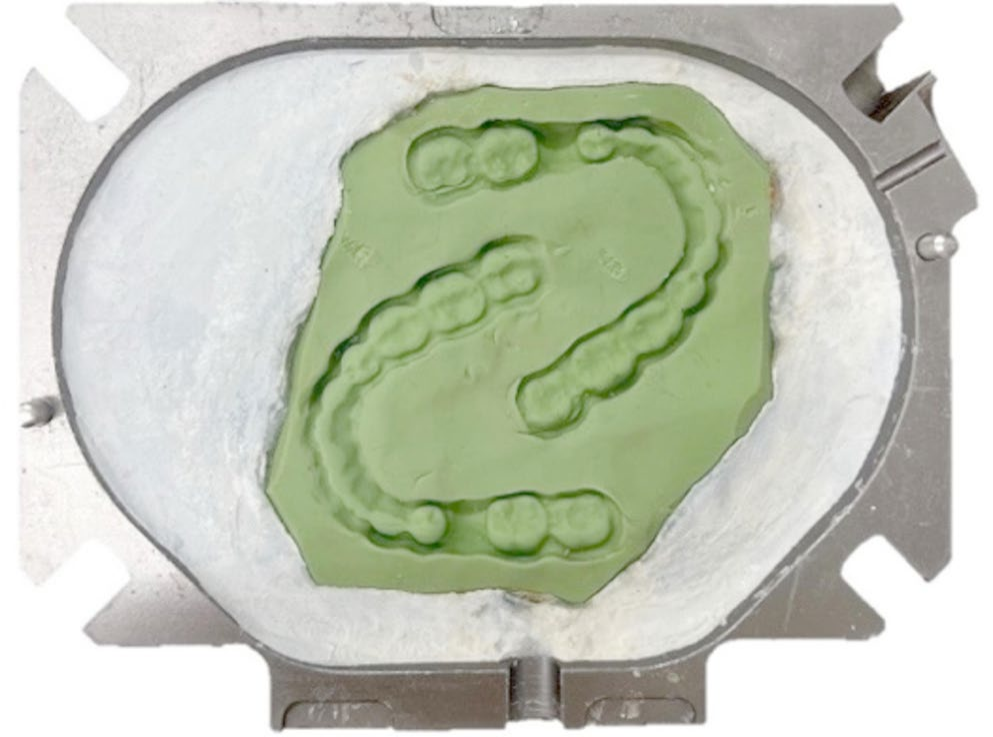
Injection technique. Occlusal device geometry embedded in a flask

The specimens were produced using the injection technique (Fig. 5). For this purpose, a splint (V-Print Splint, VOCO, Cuxhaven, Germany) was printed from the stl master data set. The base of two printed occlusal devices was pressed into silicone (Fifty-Fifty 95 putty, Klasse 4 dental, Augsburg, Germany) to obtain a precise duplicate of the geometry. The silicone mold together with the splints was embedded in a cuvette with plaster (pico-crema soft, picodent, Wipperfürth, Germany). Casting channels made of wax (PalaXpress accessory casting wax, Kulzer, Hanau, Germany) were applied. A silicone counter (Fifty-Fifty 95 putty, Klasse 4 dental) was produced, and the final layer of plaster applied. After the plaster hardened, the flask was opened and the splints removed, the flask was attached to the injection device and the material was injected. The flask remained in the injection device for 7 min and polymerization took place in the pressure pot (two bar, 55 degrees Celsius Palamat elite, Kulzer) for 35 min.

### Aging regime

All measurements were performed after 24 h of water storage after manufacturing (deionized water, 37 °C, HERAcell 150, Thermo Fisher Scientific, Waltham, USA) as well as after thermal aging (THE-1100, SD Mechatronik, Feldkirchen-Westerham, Germany) for 5,000 cycles with an immersion time of 30 s in 5 °C / 55 °C deionized water and a dwell time of 5 s.

### Test methods

**Fig. 6:**
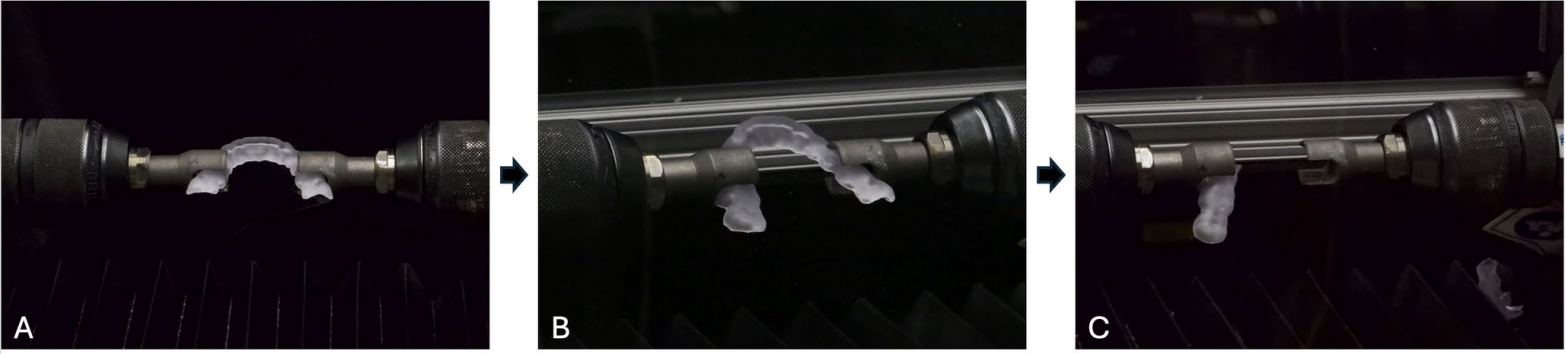
Testing device. A, Occlusal device clamped in testing device. B, Occlusal device rotating during the test. C. Fractured Occlusal device– end of the test

The specimens were investigated in a torsion testing machine (Torsion MT1-SPI, Instron, Norwood, USA). A specially designed testing device was mounted on the torsion testing machine (Fig. 6A) and each splint was clamped in the premolar region. The side of the splint containing the third quadrant was rotated counterclockwise until a crack / fracture occurred or the force significantly decreased while the splint in the fourth quadrant remained fixed in the holder without any rotation (Fig. 6B, C). The torque load (Ncm) and angular rotation (deg) were continuously monitored by the software. Fractures were classified as follows: (0) deformation, (1) crack, (2) fracture in two fracture parts, (3) complex fracture into ≥ 3 parts.

### Statistical analysis

The data were analyzed statistically with SPSS version 26.0 (IBM, SPSS, Statistics, Armonk, NY, USA). A statistical evaluation of the data was performed using descriptive analysis followed by Kolmogorov–Smirnov to test the violation of normal distribution. Parametric tests were performed, as 10% (2/20) of the TL and 0% (0/20) of the AR groups deviated from normal distribution. To determine the influence of the materials, one-way ANOVA followed by Scheffé post-hoc test was computed. A two-group *t*-test investigated the impact of the aging regimes. The fracture type distribution was analyzed by the chi-square test and Ciba-Geigy table. *p* values less than 0.05 were interpreted as statistically significant.

## Results

### Comparison of torque load of all tested materials

#### Impact of the material on torque load

bTH initially showed the lowest TL values compared to aPG, aVS, and the remaining subtractively and conventionally manufactured materials (*p* < 0.001–0.012) (Table 3). The additively manufactured materials presented lower values than the subtractively manufactured materials, except for bTH and the conventionally manufactured group (*p* < 0.001–0.029). bTP showed initially the highest TL values compared to all materials (*p* < 0.001–0.016). Within the thermal cycling group, bTH and aVC showed the lowest TL values compared to aPG and the remaining subtractively and conventionally manufactured materials (*p* < 0.001–0.009). aVS, aPP and aPG presented lower values than the subtractively manufactured materials, except for bTH and the conventionally manufactured group (*p* < 0.001). bTP showed the highest TL values compared to all materials also after thermal cycling (*p* < 0.001).

**Table 3:** Descriptive statistics (mean, standard deviation (SD) and 95% confidence intervals (CI)) for maximum torque (N*cm), angle at maximum torque (deg)

Material	Aging	torque load (TL)		angular rotation (AR)	
		Mean ± SD	95% CI	Mean ± SD	95% CI
aPG	Initial	101 ± 23.5^bA^	[75;126]	55.9 ± 7.2^abA^	[47;64]
	Thermal cycling	88.1 ± 8.8^bA^	[77;98]	54.8 ± 3.5^abA^	[50;59]
aPP	Initial	85.1 ± 28.4^abB^	[54;116]	53.7 ± 18.3^abA^	[33;73]
	Thermal cycling	58.7 ± 12.8^abA^	[44;73]	55.2 ± 13.5^abA^	[40;70]
aVS	Initial	104 ± 3.8^bB^	[99;110]	41.7 ± 2.9^abB^	[37;45]
	Thermal cycling	54.7 ± 9.6^abA^	[43;65]	19.9 ± 2.1^aA^	[16;23]
aVC	Initial	63.7 ± 6.1^abB^	[56;71]	143 ± 15.3^cB^	[126;160]
	Thermal cycling	45.7 ± 4.4*^aA^	[40;51]	123.8 ± 19.8^dA^	[102;145]
bBC	Initial	185 ± 22.0*^cB^	[161;209]	70.4 ± 8.8^bA^	[60;80]
	Thermal cycling	167 ± 10.8^cA^	[154;179]	76.4 ± 7.2^bcA^	[67;85]
bEP	Initial	161 ± 5.1^cB^	[155;168]	135 ± 28.0^cA^	[105;166]
	Thermal cycling	155 ± 4.8^cA^	[149;161]	122 ± 20.8^dA^	[99;145]
bPC	Initial	163 ± 36.3^cA^	[124;202]	38.4 ± 12.0^abA^	[24;52]
	Thermal cycling	141 ± 22.6^cA^	[116;165]	48.1 ± 6.2^abA^	[40;55]
bTP	Initial	265 ± 27.1^dA^	[235;294]	138 ± 23.9^cA^	[112;164]
	Thermal cycling	246 ± 26.9^dA^	[216;275]	131 ± 26.8^dA^	[102;160]
bTH	Initial	39.2 ± 1.7^aB^	[36;42]	115 ± 21.4^cA^	[91;138]
	Thermal cycling	31.2 ± 3.8^aA^	[26;36]	111 ± 32.8^cdA^	[75;146]
cPB	Initial	204 ± 26.4^cB^	[176;233]	29.3 ± 4.2^aB^	[23;34]
	Thermal cycling	138 ± 18.5^cA^	[118;159]	19.5 ± 3.1^aA^	[15;23]

* Deviation from the normal distribution

abcd Different small letters indicate significant differences between the materials within one aging regime

AB Different capital letters indicate significant differences between the aging regimes within one material

CI Confidence interval

SD Standard deviation

#### Impact of artificial aging on torque load

Artificial aging affected the TL values of all materials except for aPG, bPC and bTP (*p* = 0.112–0.127) (Table 3). Thermal cycling reduced the TL values for the remaining materials (aPP, aVS, aVC, bBC, bEP, bTH and cPB) (*p* < 0.001–0.049).

### Comparison of angular rotation of all tested materials

#### Impact of the material on angular rotation

cPB showed initially lower AR values compared to bBC, bTH, bEP, bTP and aVC (*p* < 0.001–0.047) (Table 3). bTH, bEP, bTP and aVC presented the highest AR values (*p* < 0.001–0.019). Within the thermal cycling group cPB and aVS showed initially lower AR values compared to bBC, bTH, bEP, aVC and bTP (*p* < 0.001–0.001).

bTH, bEP, aVC and bTP showed the highest AR values also after thermal cycling (*p* < 0.001–0.025).

#### Impact of artificial aging on angular rotation

Artificial aging affected the AR values of the materials except for aPG, aPP and subtractively manufactured group (*p* = 0.055–0.434) (Table 3). Thermal cycling reduced the AR values for the remained materials (aVS, aVC and cPB) (*p* < 0.001–0.040).

### Comparison of fracture types of all tested materials

**Fig. 7:**
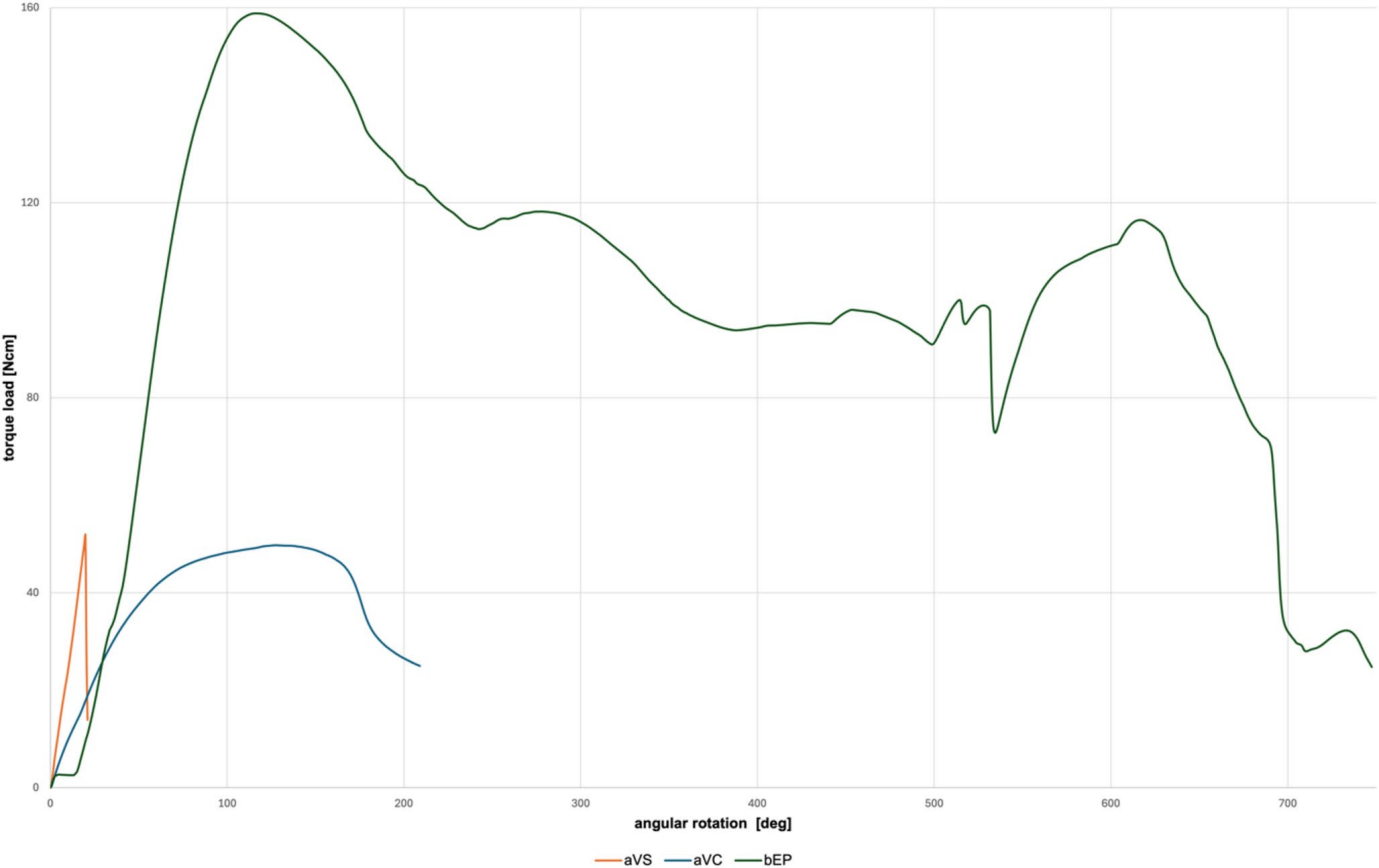
Torque load / angular rotation curves of the thermal cycling group for the materials aVS (fracture in two parts), aVC (crack) and bEP (deformation)

Following four fracture types were evaluated: deformation, crack, fracture in two parts and complex fracture into ≥ three parts. 95% CI and percentage of investigated fracture types are summarized in Table 4. For aPG, aPP, bBC, bPC, and cPB predominantly fractures in two parts were observed initially and after thermal cycling (66.67–100%). aVS showed initially 100% complex fractures, while thermal cycling led to 100% fractures into two parts. Fractures into two parts were observed initially for aVC and bTP (50-66.67%), while after thermal cycling, mostly cracks occurred for these materials (66.67–100%). Deformation was shown initially and after thermal cycling for bEP and bTH (83.33–100%). The different fracture behavior can be observed in the torque load / angular rotation diagram (Fig. 7).

**Table 4:** Fracture type distribution [(0) deformation, (1) crack, (2) fracture in two fracture parts, (3) complex fracture into ≥ 3 parts] of the specimens (%) with 95% confidence interval (CI)

Material	Fracture type	Initial		Thermal cycling	
		*n*	95% CI	*n*	95% CI
aPG	0	0	[0;46]	0	[0;46]
	1	0	[0;46]	0	[0;46]
	2	100	[53;100]	100	[53;100]
	3	0	[0;46]	0	[0;46]
aPP	0	0	[0;46]	0	[0;46]
	1	0	[0;46]	0	[0;46]
	2	83.33	[34;100]	100	[53;100]
	3	16.67	[0.3;65]	0	[0;46]
aVS	0	0	[0;46]	0	[0;46]
	1	0	[0;46]	0	[0;46]
	2	0	[0;46]	100	[53;100]
	3	100	[53;100]	0	[0;46]
aVC	0	0	[0;46]	0	[0;46]
	1	50	[10;89]	100	[53;100]
	2	50	[10;89]	0	[0;46]
	3	0	[0;46]	0	[0;46]
bBC	0	0	[0;46]	0	[0;46]
	1	0	[0;46]	0	[0;46]
	2	66.67	[21;96]	66.67	[21;96]
	3	33.33	[4.2;78]	33.33	[4.2;78]
bEP	0	100	[53;100]	83.33	[34;100]
	1	0	[0;46]	0	[0;46]
	2	0	[0;46]	16.67	[0.3;65]
	3	0	[0;46]	0	[0;46]
bPC	0	0	[0;46]	0	[0;46]
	1	0	[0;46]	0	[0;46]
	2	100	[53;100]	100	[53;100]
	3	0	[0;46]	0	[0;46]
bTP	0	16.67	[0.3;65]	33.33	[4.2;78]
	1	16.67	[0.3;65]	66.67	[21;96]
	2	66.67	[21;96]	0	[0;46]
	3	0	[0;46]	0	[0;46]
bTH	0	100	[53;100]	100	[53;100]
	1	0	[0;46]	0	[0;46]
	2	0	[0;46]	0	[0;46]
	3	0	[0;46]	0	[0;46]
cPB	0	0	[0;46]	0	[0;46]
	1	0	[0;46]	0	[0;46]
	2	100	[53;100]	100	[53;100]
	3	0	[0;46]	0	[0;46]

## Discussion

The aim of this investigation was to examine the torsional resistance of four additively, five subtractively and one conventionally manufactured materials for occlusal devices. The tested null hypotheses stating that neither the choice of material nor artificial aging procedure showed a significant impact on torque load (TL) and angular rotation (AR) were rejected.

After all aging regimes, bTP displayed the highest TL values, followed by the conventionally manufactured material cPB and the remaining subtractively manufactured materials, with the exception of bTH. This is supported by previous investigations, where bTP showed the highest flexural strength values [10, 13]. Initially, only 66.67% of the bTP specimens demonstrated fracture, while no fractures were observed after thermal cycling. The aged bTP specimens exhibited either cracks or deformation, but not complete failure. These findings align with previous studies investigating the flexural strength where bTP showed no fracture behavior attributed to the ductility of Polycarbonate (PC) based materials, which enhances its ability to withstand high masticatory forces without fracturing [10, 13, 34]. Previous investigations of bTP showed high values for fracture toughness and work of fracture, reflecting the material’s capacity for plastic deformation and a high amount of energy required to induce fracture [6, 13]. However, further clinical studies are necessary to investigate whether more resilient PC-based materials are an option for occlusal devices where high bite forces occur, such as in patients with severe bruxism. The present study found no significant difference in the TL values between conventionally and subtractively manufactured PMMA-based materials, which aligns with previous studies that reported no significant differences in the flexural strength [5, 6] and elastic modulus values of these materials [10]. However, this result contrasts with an earlier investigation where conventionally manufactured PMMA-based materials showed lower flexural strength values compared to subtractively manufactured PMMA, attributed to improved material properties such as a more homogeneous, highly cross-linked structure and reduced porosity of the blanks [10, 35, 36]. These findings highlight the need for further research to explore the relationship between torque load and associated mechanical properties such as flexural strength and fracture toughness. Initially and after thermal cycling, all 3D-printed materials, as well as the subtractively manufactured bTH, showed the lowest TL values. These results are consistent with earlier investigations where 3D-printed materials showed lower mechanical properties compared to both subtractively and conventionally manufactured materials [6, 15, 16, 36, 37]. This can be attributed to the higher water sorption values of 3D-printed resins. Microscopic voids between the resin layers during the printing process as well as lower DC values contribute to this effect [10, 21, 38]. This could compromise the material’s structural integrity and lead to reduced mechanical properties such as torque resistance. In contrast, another study observed significantly higher flexural strength values for aVS compared to aPG, aPP, and aVC, with values exceeding 65 MPa [10]. However, another study documented that aVS exhibited a flexural strength of only 37 MPa [36]. Different printing orientation, layer thickness and postpolymerization significantly affect the mechanical properties, highlighting the different results in the investigations [14, 15, 18, 21]. bTH showed the lowest TL values among all tested materials. Interestingly, despite its lower torque resistance, bTH was the only material that did not show any cracks or fracture under torsional stress, either initially or after thermal cycling. bTH showed deformation under the applied load, meaning it maintained its structural integrity without catastrophic failure. This behavior indicates that Polyethylmethacrylat based materials are highly resistant to fracture, even though it exhibits lower TL values than other materials. The material’s ability to undergo plastic deformation without fracture, despite its lower TL, reflects its toughness and flexibility. However, the combination of low torque resistance and high plastic deformability might also suggest that bTH has a lower hardness. A previous investigation already showed low HM values for bTH [10]. In literature, a correlation between hardness and wear resistance is observed. Materials with lower hardness are typically more prone to wear over time, especially when subjected to abrasive forces such as grinding [4, 15, 16]. Therefore, it is critical to investigate whether the high degree of plasticity observed in bTH leads to increased material wear during long-term use.

The 3D-printed material aVC, as well as the subtractively manufactured materials bTP, bEP, and bTH, exhibited the highest angular rotation values both initially and after thermal cycling. These were also the only materials that predominantly demonstrated deformation and cracks. Only 50% of the aVC specimens fractured into two parts initially, and 16,67% of the bEP specimens fractured after thermal cycling. Since bEP showed 100% deformation initially and 83,33% after aging, the 16,67% fracture rate post-aging could be attributed to material defects. In contrast, the additively manufactured materials aPG, aPP, and aVS, along with the subtractively manufactured materials bBC and bPC, and the conventionally manufactured material cPB, demonstrated consistently low angular rotation values both initially and after aging. All these specimens exhibited fracture behavior in two parts or complex fracture. In a previous study, the materials aPG, aPP, and bBC did not fracture during flexural strength testing [10]. This discrepancy can be attributed to differences in the test method, as the previous study limited specimen deflection to 10 mm until test termination, whereas the torsional setup used in the current investigation permitted a higher extent of material deformation. The PMMA-based materials bPC and cPB showed the lowest initial angular rotation values, likely due to their brittleness and low ductility [10]. Thermal cycling resulted in a substantial reduction in the angular rotation of aVS, with the fracture pattern becoming more complex, as evidenced by an increase in the number of fragments from two to complex fracture after aging.It has been observed that the flexural strength and elastic modulus of aVS decreased after aging, while water sorption increased after 90 days of water storage and both water solubility and the degree of conversion increased after 90 days of water storage and thermal cycling [10]. These changes may have contributed to an increase in material brittleness, which could explain both the higher number of fractured specimens and the reduced angular rotation observed in the present study after thermal cycling.

The materials aPG, bPC, and bTP showed no impact of aging on TL and AR. Furthermore, aging did not influence AR of the materials aPP, bBC, bEP, bPC, and bTH. The remaining materials exhibited a decrease in both TL and AR after aging, which is consistent with a previous investigation, where the mechanical properties decreased after aging [17].

Since patients may wear the occlusal devices up to 23 h daily, thermal cycling tests are relevant, with 5,000 cycles representing six months in vitro [38–40]. However, a limitation of this study is it´s in vitro design, which does not fully simulate clinical conditions, as the rotational movements tested do not occur to the same extent when the splint is worn in the oral environment.

The findings of this study highlight the diverse material properties of dental splints produced by different manufacturing methods. The current literature shows a variability on whether softer, more flexible splints or harder, rigid splints are more effective in alleviating symptoms in patients with craniomandibular dysfunction (CMD) [5, 25–31, 41, 42]. While softer splints may offer improved comfort and adaptability, harder splints might provide superior occlusal stabilization and durability. This disparity underlines the need for clinical studies to identify the material characteristics that optimize both pain reduction and functional outcomes for CMD patients. Further research should focus on systematically evaluating the correlation between material properties, patient comfort and the long-term durability of occlusal devices. Such studies would help establish evidence-based guidelines for selecting the most appropriate occlusal device for individual patient needs.

## Conclusion

Within the limitations in this in vitro study, the following conclusions were drawn:

1. The composition of the material has a greater impact on torsional resistance than the manufacturing method, as significant differences were observed within the subtractively manufactured group.
2. All additively manufactured materials showed similar TL values, while aVC showed significantly higher AR values.
3. Only the materials aVS, aVC, bTH, and cPB showed a decrease in both TL and AR values after aging.
4. Material selection for occlusal devices should be based on the patient’s clinical needs, considering their mechanical properties.

## Data Availability

No datasets were generated or analysed during the current study.
